# Systemic analysis of gene expression profiles in porcine granulosa cells during aging

**DOI:** 10.18632/oncotarget.21731

**Published:** 2017-10-10

**Authors:** Li Hui, Guo Shuangshuang, Yu Jianning, Shi Zhendan

**Affiliations:** ^1^ Key Laboratory of Animal Breeding and Reproduction, Institute of Animal Science, Jiangsu Academy of Agricultural Sciences, Nanjing, China

**Keywords:** granulosa cell, aging, porcine, gene expression, RNA-seq, Gerotarget

## Abstract

Current studies have revealed that aging is a negative factor that suppresses granulosa cell functions and causes low fertility in women. However, the difference in gene expression between normal and aging granulosa cells remains undefined. Therefore, the aim of this study was to investigate the gene expression profiles of granulosa cells during aging. Granulosa cells from young healthy porcine ovaries were aged *in vitro* by prolonging the culture time (for 48h). First, the extracellular ultrastructure was observed by scanning electron microscopy followed by RNA-seq and KEGG pathway analysis. The results showed that the extracellular ultrastructure was significantly altered by aging; cell membranes were rough, and cavitations were found. Moreover, the formations of filopodia were greatly reduced. RNA-seq data revealed that 3411 genes were differentially expressed during aging, of which 2193 genes were up-regulated and 1218 genes were down-regulated. KEGG pathway analysis revealed that 25 pathways including pathway in cancer, PI3K-Akt signaling pathway, focal adhesion, proteoglycans in cancer, and cAMP signaling pathway were the most changed. Moreover, several high differentially expressed genes (CEBPB, CXCL12, ANGPT2, IGFBP3, and BBOX1) were identified in aging granulosa cells, The expressions of these genes and genes associated with extracellular matrix remodeling associated genes (TIMP3, MMP2, MMP3, and CTGF), energy metabolism associated genes (SLC2A1, PPARγ) and steroidogenesis associated genes (StAR, CYP11A1 and LHCGR) were confirmed by quantitative PCR. This study identifies the differently changed pathways and their related genes, contributes to the understanding of aging in granulosa cells, and provides an important foundation for further studies.

## INTRODUCTION

In human females, reproductive organs age more rapidly than other parts of the body [1]; women older than 35 years typically exhibit low fertility [2]. The decline in fecundity accelerates from 35 to 40 years, and fertility is almost zero at 45 years [3]. In developed countries, women tend to delay having their first child, resulting in decreased birth rates and a more aged population, which is becoming a serious social problem [4]. Studies have shown that poor occyte quality caused by aging is the main reason for low fertility in elderly women [5-8].

Oocyte quality is strongly influenced by the microenvironment provided by granulosa cells (GCs) [9]. It is well-known that oocytes are separated from the blood by the surrounding GCs, follicular fluid, and theca cells. Hence, they are unable to obtain nutrients directly from the blood during development, 85 % of nutrients including hormones, growth factors, amino acids, and energy sources are supplied by GCs [10-17]. In addition, GCs also regulate the transcriptional activity of oocytes and facilitate the post-transcriptional modification of several oocyte proteins [18]. Interestingly, previous studies have demonstrated that the quality of oocytes could be improved following their isolation from aged mice follicles and maturation *in vitro* [19]. Moreover, aging GCs could also accelerate oocyte aging [20-24]. Taken together, these results suggest that oocyte quality is affected by deteriorating GCs [25].

Aging has a negative effect on GC functions [26]. In elderly women, the apoptosis ratio of GCs is significantly increased [27], moreover, the decrease in superoxide dismutase (SOD1 and SOD2), coenzyme Q10, and catalase were decreased along with defective mitochondria and reduced lipid droplets [24, 28]. The integrity of DNA in GCs is also affected by aging, moreover, a marked increase in double-strand breaks (DSBs) and a marked decrease in DSBs repair-related genes have been reported [29]. In addition, epigenetics changes were also the symptoms of the aging GCs. The GCs from old cows were found to have low genomic global DNA methylation, reduced proliferative and telomerase activity, and shortened telomeres [30]. Furthermore, the protein expression of GCs was reported to be different between young (20-33 years) and elderly (40-45 years) women, and 110 (7.7 % in total) proteins were found to be differentially expressed in aging GCs [31]. These results demonstrated that aging impairs GC functions; however, the mechanisms of aging and the gene expression profiles of aging GCs remain unclear.

Because of ethical constraints, the number of samples available for study is limited, preventing thorough investigations. Several studies have examined human GCs derived from patients undergoing *in vitro* fertilization (IVF). However, we believe that these cells do not accurately reflect actual conditions because they are derived from patients who suffer from infertility or some other illness that impairs fertility; moreover, the cells have been exposed to high pharmacological concentrations of exogenous gonadotropins during the process of super-ovulation. Therefore, the purpose of this study was to use porcine GCs as a model to evaluate the gene expression profiles during aging by RNA-seq and to understand the causes of fecundity declines in elderly women.

## RESULTS

### Ultrastructure changes in aging GCs

**Figure 1:**
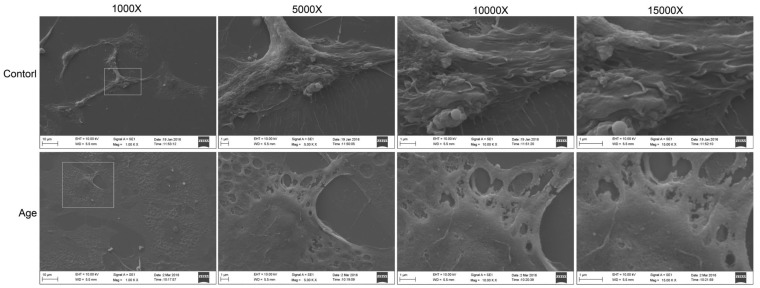
Extracellular ultrastructure analysis using scanning electron microscope Ultrastructures observed at 1000×, 5000×, 10000×, and 15000×, respectively.

Various studies have investigated the intracellular ultrastructure changes in aging GCs by transmission electron microscopy and found defective mitochondria and reduced lipid droplets in the cytoplasm [28]. In the present study, to elucidate the effects of aging on GCs, changes in the cellular morphology and extracellular structure (especially the cell membrane) were analyzed by scanning electron microscopy. As shown in Figure 1, the cell membrane surface was rough, cavitations were found, and the formation of filopodia was greatly reduced in aging GCs compared with the control GCs. Moreover, cells in the aging group appeared flat. These results showed that aging could disrupt cell membrane integrity and fluidity.

### Overview of sequencing data

**Figure 2:**
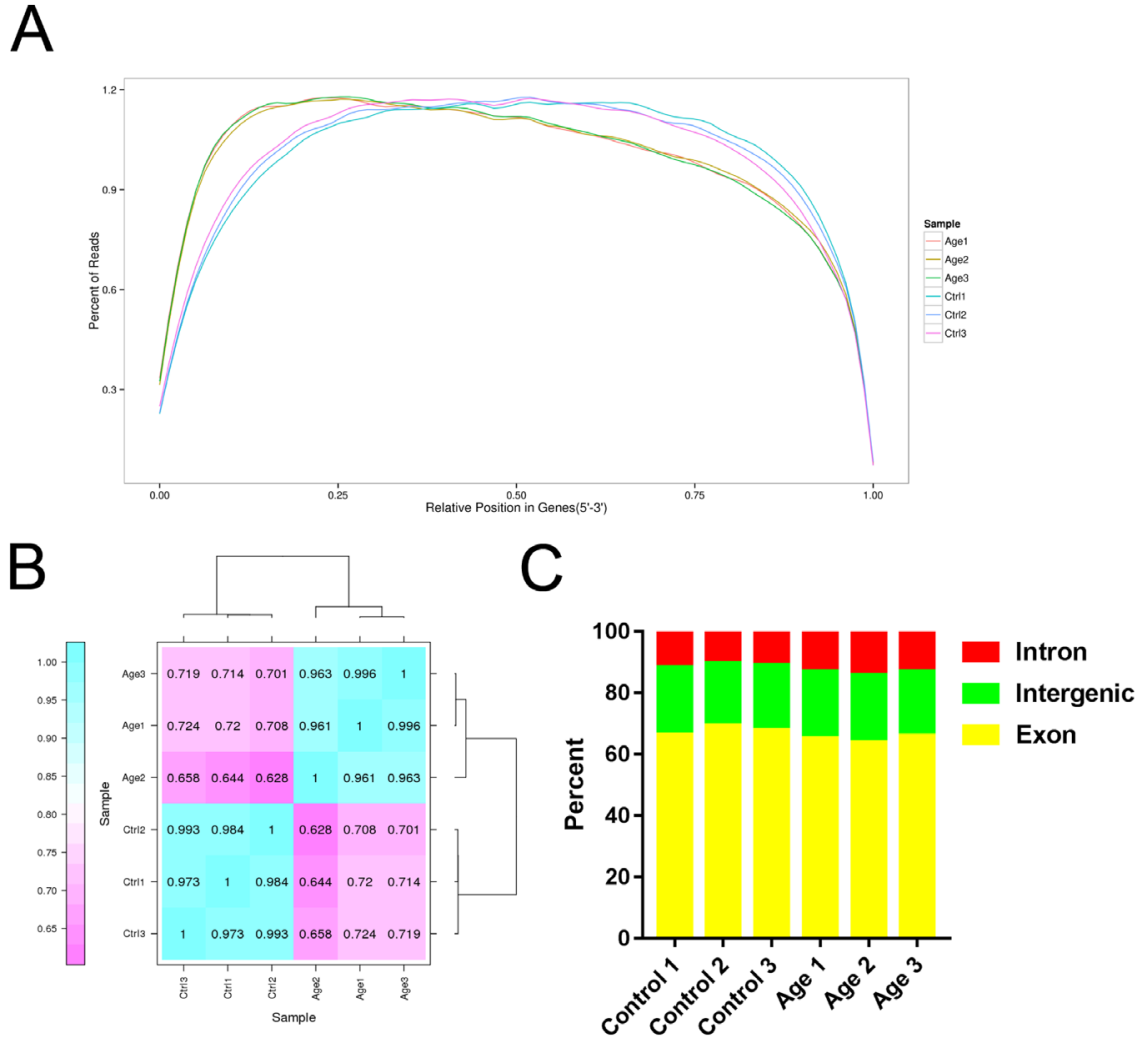
Overview of RNA sequencing data **A.** The distribution of mapped reads on mRNA (5′-3′). Data in reflects the percentage of mapped reads assigned to all regions of mRNA. The location of the normalized mRNA is on the horizontal (x) axis; the percentage of reads as compared to total mapped reads for the position is on the vertical (y) axis. **B.** Heat map of the biological replicates. **C.** Reads mapped to different regions of the gene.

After RNA-seq analysis, we obtained 51.84 Gb of clean data from six libraries. A total of 64809040, 64799480, 73957792, 54410404, 65145146 and 56386430 clean reads were obtained from the respective libraries after filtering low-quality reads and removing adaptor sequences, and approximately 81.89% of the total clean reads were mapped to the *Sus scrofa* genome assembly in NCBI (http://www.ncbi.nlm.nih.gov/genome/?term = Sus_scrofa). In addition, the Q30 ranged between 90.76% and 93.06%. These results indicated that the quality of our six libraries was good, thus they were suitable for analysis. The detected number of genes was 22293, 22851, 22662, 25141, 23789 and 23974 for the respective libraries (Table 1). The vast majority of reads were evenly distributed throughout the gene by random sampling, which indicated the quality and homogeneity of the samples (Figure 2A). The samples collected form three separated experiments were good biological replicates (Figure 2B). Moreover, the ratio of reads corresponding to the exon, intron, and intergenic regions was different (Figure 2C). In total, 23159 genes were annotated (Table 2) and 3411 genes were differentially expressed in GCs during aging, of which 2193 genes were up-regulated and 1218 genes were down-regulated (Detailed information was shown in Supplementary Files 1 and 2). The top 10 up-regulated and down-regulated genes are shown in Table 3. Moreover, 2210 new genes were detected in the analysis, and 1580 genes were annotated in a different database (Table 2) (Detail information was shown in Supplementary File 3).

**Table 1:** Basic characteristics of RNA-seq data in porcine GCs during aging.

	Biological replicate 1		Biological replicate 2		Biological replicate 3	
Type	Control 1	Aging 1	Control 2	Aging 2	Control 3	Aging 3
Total Raw Reads	65275748	54539100	64997294	65296682	74624954	56512978
Total Clean Reads	64809040	54410404	64799480	65145146	73957792	56386430
Total Clean Reads Ratio(%)	99.29	99.76	99.70	99.77	99.11	99.78
Clean Reads Q30(%)	90.76	92.91	91.84	93.06	91.15	92.84
Total mapped reads	84.28%	79.38%	85.13%	79.33%	83.96%	79.27%
Unique mapped reads	78.36%	74.01%	79.12%	74.03%	78.06%	73.91%
Reads mapped in paired	78.69%	71.35%	80.11%	71.44%	78.46%	71.07%
Read mapped Gene	55.51%	53.60%	55.42%	55.98%	55.99%	54.34%
Detected Gene Number	22293	25141	22851	23789	22662	23974
Reads Map to '+'	25,874,673 (39.92%)	20,433,831 (37.56%)	26,226,379 (40.47%)	24,457,340 (37.54%)	26,211,265 (40.45%)	21,144,814 (37.50%)
Reads Map to '-'	25,848,028 (39.88%)	20,456,183 (37.60%)	26,211,265 (40.45%)	24,463,039 (37.55%)	29,430,847 (39.79%)	21,165,749 (37.54%)

Triplicate samples were collected from separated experiments. GC (%) is the percentage of G and C bases / total nucleotides; *N* (%) is the percentage of unrecognized bases / total nucleotides; Q30 (%) is the percentage of bases’ mass greater than or equal Q30 in the clean data; Uniq Mapped Reads is the number and percentage of reads that mapped to a unique position in the reference genome in the clean reads; Multiple Mapped Reads is the number and percentage of reads that mapped to multiple positions in the reference genome in the clean reads; Reads Map to ‘+’ is the number of clean reads mapped to the sense strand; Reads Map to ‘−’ is the number of clean reads mapped to antisense strand.

**Table 2:** Annotated gene numbers and length distribution in different databases

Annotation Database	Annotated Number (All Genes/New Genes)	300<=length<1000 (All Genes/New Genes)	length>=1000 (All Genes/New Genes)
COG Annotation	7016/194	2554/70	4308/118
GO Annotation	20881/813	8826/298	10935/478
KEGG Annotation	13324/593	5295/214	7438/347
Swiss-Prot Annotation	14455/771	5441/226	8253/521
Nr Annotation	19735/1576	8241/510	10603/1007
All Annotated	23159/1580	9897/512	11960/1009

COG : Clusters of Orthologous Groups; GO: Gene Ontology; KEGG: Kyoto Encyclopedia of Genes and Genomes; Nr: Non-Redundant

**Table 3:** Top ten up-regulated and down-regulated genes in porcine GCs during aging

Gene Name	Description	Log2 Fold Change	P-Value
Up-regulated			
CEBPB	CCAAT/enhancer binding protein beta	8.012054	5.69E-94
RLN2	Relaxin 2	6.982068	5.68E-24
PGM5	Phosphoglucomutase 5	6.347641	8.96E-20
PLIN4	Perilipin 4	6.305554	1.02E-26
CHST9	Carbohydrate sulfotransferase 9	5.920979	9.32E-33
NRSN1	Neurensin 1	5.552856	9.80E-12
SCARA5	Scavenger receptor class A member 5	5.537797	3.84E-57
HKDC1	Hexokinase domain containing 1	5.346571	1.20E-24
PPP1R3G	Protein phosphatase 1 regulatory subunit 3G	5.337344	2.30E-26
CXCL12	C-X-C motif chemokine ligand 12	5.287672	6.19E-25
Down-regulated			
TNFRSF11B	TNF receptor superfamily member 11b	-6.22999	1.15E-15
IGFBP3	Insulin like growth factor binding protein 3	-5.1116	0
CXCL9	C-X-C motif chemokine ligand 9	-4.70226	6.44E-08
ANGPT2	Angiopoietin 2	-4.65853	4.29E-14
BBOX1	Gamma-butyrobetaine hydroxylase 1	-4.59239	4.77E-09
SERPINB9	Serpin family B member 9	-4.44572	1.60E-08
AREG	Amphiregulin	-4.34269	2.54E-184
TKTL1	Transketolase like 1	-4.10354	2.56E-07
RU2B	U2 small nuclear ribonucleoprotein B	-3.85723	6.94E-10
CXCL10	C-X-C motif chemokine ligand 10	-3.83186	2.89E-05

**Figure 3:**
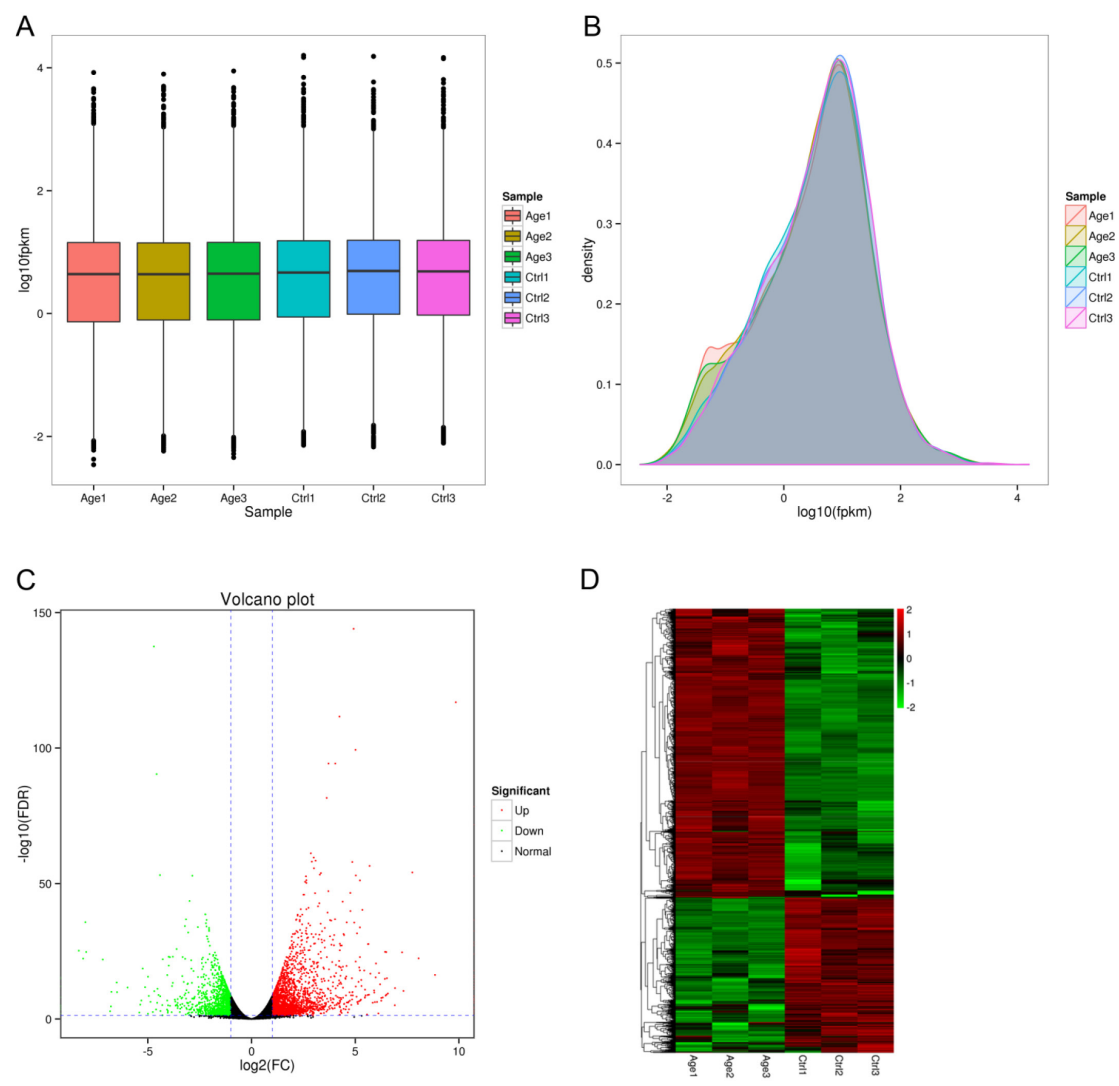
Overview of differentially expressed genes **A.** The number of differentially expressed mRNAs by sample. **B.** FPKM distributions in each sample. **C.** Volcano plot of genes differentially expressed in each sample. The log2 fold change difference is represented on the x-axis and negative log of FDR is represented on the y-axis. Each point represents a gene which had detectable expression in the 6 samples. The significant up-regulated genes are plotted in red on the right side and down-regulated genes plotted in green on the left side. The no significant differentially expressed genes were shown in dark points in the middle and the bottom. **D.** Expression differences are shown as different colors. Red indicates up-regulated while green indicates down-regulated gene expression.

Based on the FPKM, six samples showed a similar expression level, and most of the mRNAs detected in RNA-seq were from protein-coding genes (Figure 3A and 3B). Additionally, volcano plots representing the differential expression of genes were drawn, in which, gene expression ratios and the significance of differential gene expression are displayed on the X and Y axis, respectively (Figure 3C). The results of unsupervised hierarchical clustering of the differentially expressed genes (DEGs) are presented in a heat map which showed a clear discrimination between the two groups (Figure 3D).

### Gene ontology (GO) and KEGG pathway enrichment analysis

**Figure 4:**
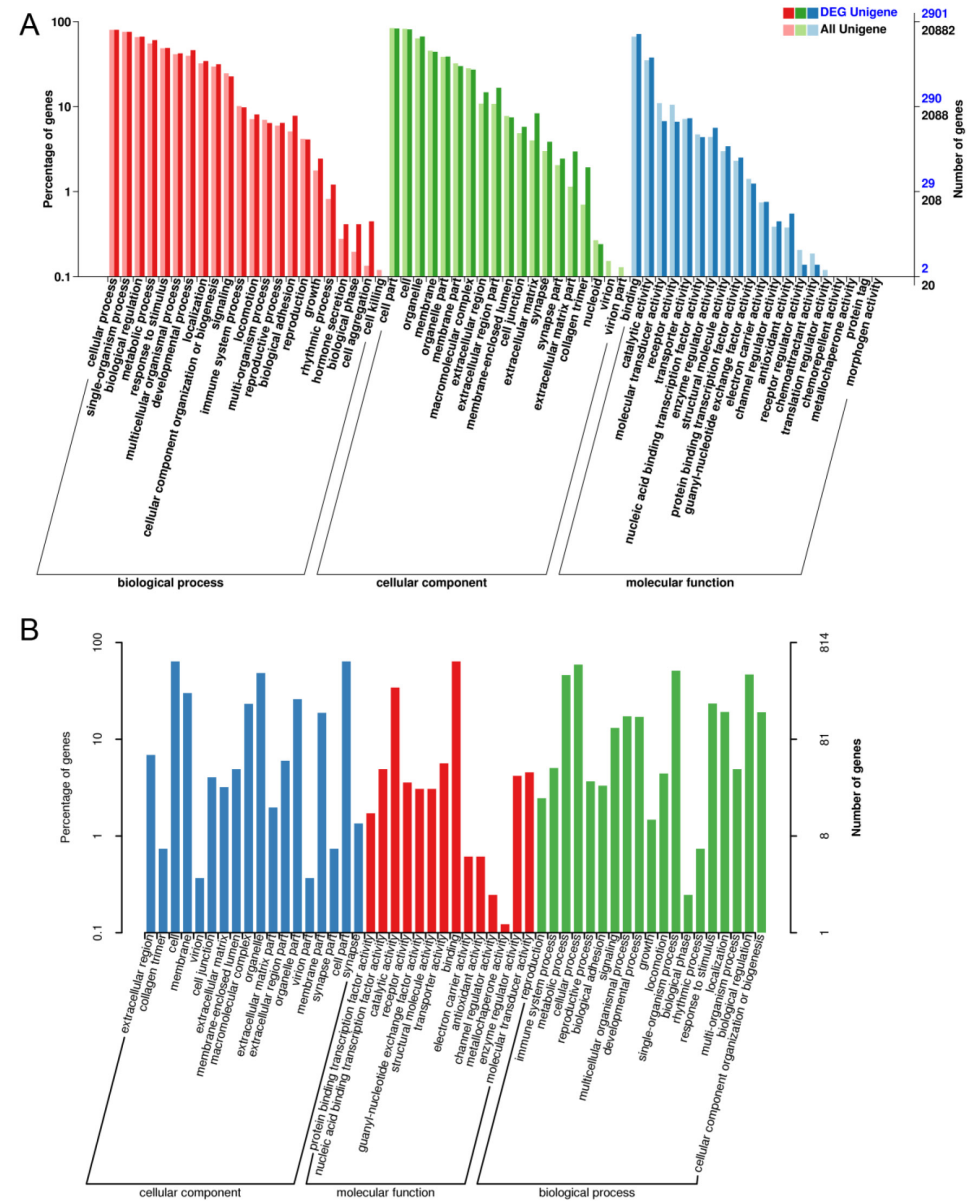
The differential expression of mRNA in aged GCs was analyzed by Gene Ontology (GO) annotation **A.** GO analysis of differentially expressed genes in aged GCs. DEG Unigene: differentially expressed genes number in all annotation Biological Process GO term. All Unigene: Unigene number in all annotation Biological Process GO term. **B.** GO analysis of new detected genes.

The most enriched GO terms for DEGs are shown in Figure 4A (Total information was listed in Supplementary File 4). The most enriched GO terms for newly identified DEGs are shown in Figure 4B. GO analyses of the newly identified DEGs were also performed (As shown in Supplementary File 4).

**Figure 5:**
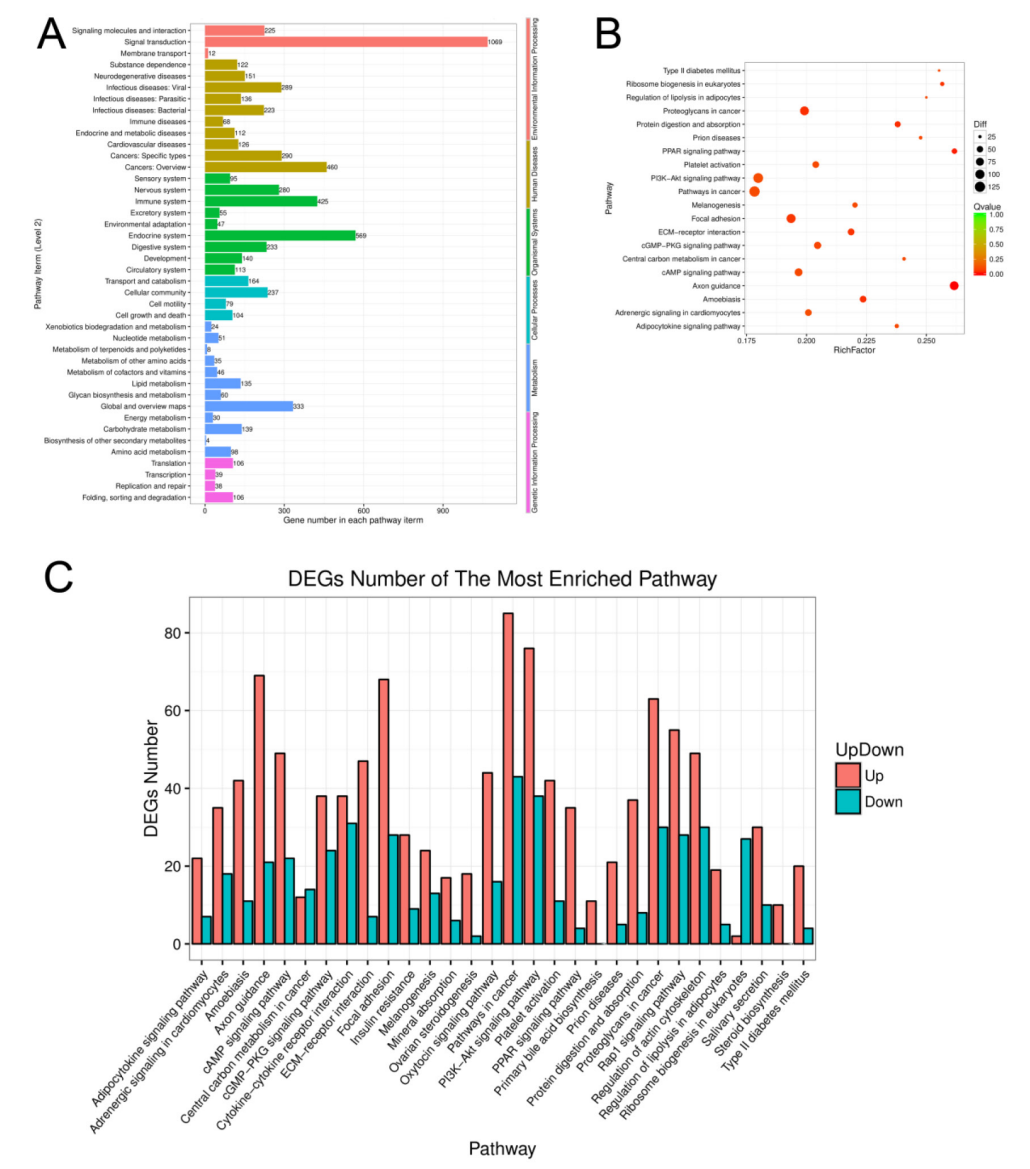
Function analysis of the differentially expressed genes of aging GCs **A.** KEGG pathway enrichment analysis of differentially expressed genes from aging GCs. **B.** Pathway enrichment analysis among DEGs. Different colors indicate different enrichment factors. The size of the plot corresponds to the number of genes. **C.** The numbers of up- and down-regulated genes in the most enrichment pathways.

A total of 1818 DEGs were assigned to KEGG annotations (Figure 5A), of which 25 enriched pathways were significantly changed (*P* < 0.01) including the pathway in cancer, PI3K-Akt signaling pathway, focal adhesion, proteoglycans in cancer, and cAMP signaling pathway (as shown in Figure 5B and Table 4.), and the numbers of up-regulated and down-regulated DEGs in most enriched pathways were shown in Figure 5C and Supplementary File 5.

**Table 4:** All the significantly changed pathways in porcine GC during aging (P<0.01)

Pathway	DGEs in term	All genes in term	Rich Factor	P-value	Q-value	Pathway ID
Pathways in Cancer	128 (4.9%)	718 (3.98%)	0.178272980501393	0.006292627	1.031292e-01	ko05200
PI3K-Akt signaling pathway	114 (4.36%)	634 (3.51%)	0.17981072555205	0.007344795	1.070215e-01	ko04151
Focal adhesion	96 (3.67%)	496 (2.75%)	0.193548387096774	0.001518009	5.597658e-02	ko04510
Proteoglycans in Cancer	93 (3.56%)	467 (2.59%)	0.199143468950749	0.0007058003	4.164222e-02	ko05205
cAMP signaling pathway	71 (2.72%)	361 (2%)	0.196675900277008	0.003890199	9.302484e-02	ko04024
Adrenergic signaling in cardiomycytes	53 (2.03%)	264 (1.46%)	0.200757575757576	0.007605771	1.070215e-01	ko04261
cGMP-PKG signaling pathway	62(2.37%)	303(1.68%)	0.204620462046205	0.00264789	8.679198e-02	ko04022
Platelet activition	53 (2.03%)	260 (1.44%)	0.203846153846154	0.005571454	9.668111e-02	ko04611
ECM-receptor interaction	54 (2.07%)	247 (1.37%)	0.218623481781377	0.001064528	5.116876e-02	ko04512
Melanogenesis	37 (1.42%)	168 (0.93%)	0.220238095238095	0.005287348	9.668111e-02	ko04916
Amoebiasis	53 (2.03%)	237 (1.31%)	0.223628691983122	0.0006811799	4.164222e-02	ko05146
Protein digestion and absorption	45 (1.72%)	189 (1.05%)	0.238095238095238	0.000412861	4.059800e-02	ko04974
Adipocytokine signaling pathway	29 (1.11%)	122 (0.68%)	0.237704918032787	0.004151216	9.302484e-02	ko04920
Central carbon metabolism in cancer	26 (1%)	108 (0.6%)	0.240740740740741	0.00537483	9.668111e-02	ko05230
Prion diease	26 (1%)	105 (0.58%)	0.247619047619048	0.00359846	9.302484e-02	ko05020
Regulation of lipolysis in adipocytes	24 (0.92%)	96 (0.53%)	0.25	0.004414738	9.302484e-02	ko04923
Ribosome biogenesis in eukaryotes	29 (1.11%)	113 (0.63%)	0.256637168141593	0.001214174	5.116876e-02	ko03008
Type II diabetes mellitus	24 (0.92%)	94 (0.52%)	0.25531914893617	0.003302304	9.302484e-02	ko04930
PPAR signaling pathway	39 (1.49%)	149 (0.83%)	0.261744966442953	0.0001245343	1.836881e-02	ko03320
Axon guidance	90 (3.44%)	344 (1.91%)	0.261627906976744	7.418738e-09	2.188528e-06	ko04360
Ovarian steroidogenesis	20 (0.77%)	79 (0.44%)	0.253164557	0.007657	1.07E-01	ko04913
Oxytocin signaling pathway	60 (2.3%)	306 (1.69%)	0.196078431	0.007981	1.07E-01	ko04921
Salivary secretion	40 (1.53%)	190 (1.05%)	0.210526316	0.008535	1.09E-01	ko04970

### Validation of representative genes by QRT-PCR

**Figure 6:**
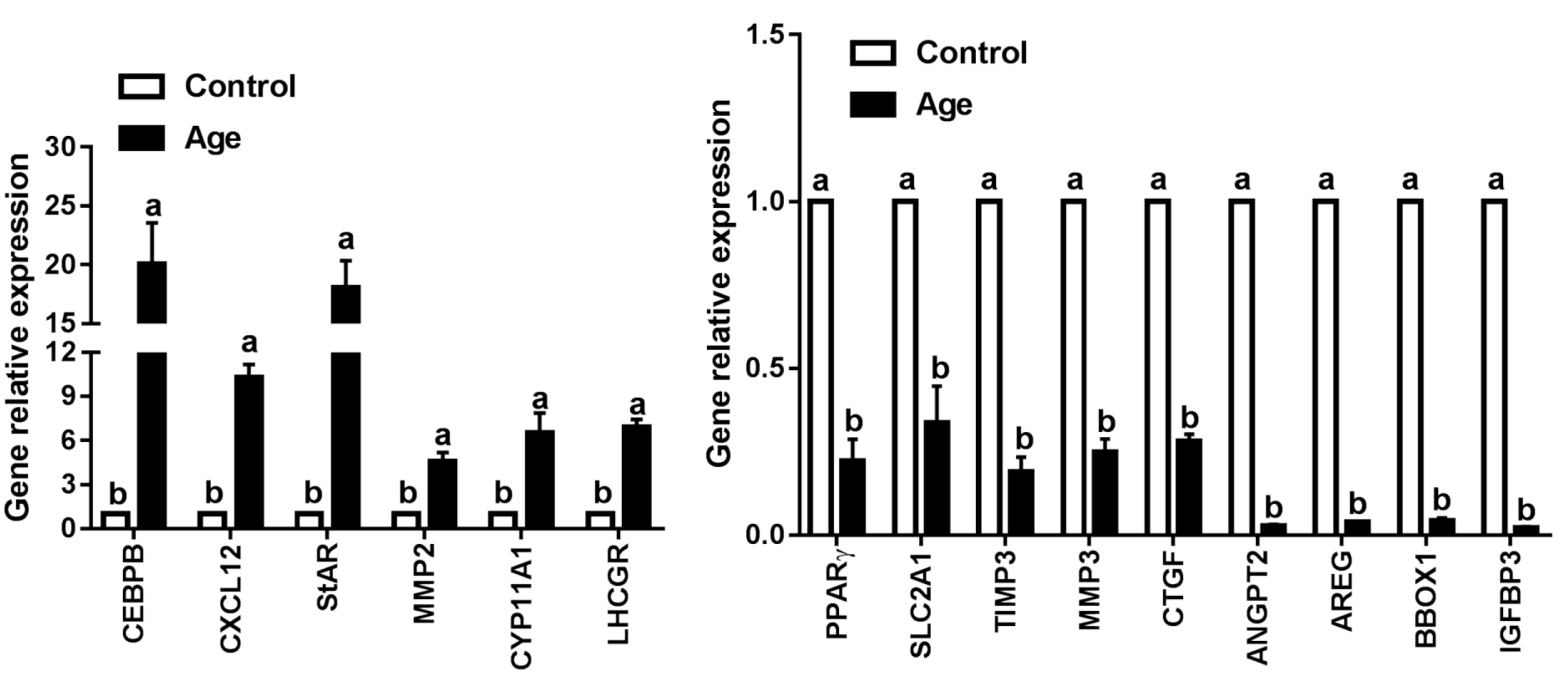
Validation the interested genes identified by RNA-seq using QRT-PCR QRT-PCR was performed to make comparisons between control and aging GCs. Results are expressed as the mean ± SEM of at least 3 independent experiments and values with different letters are significantly different (*P* < 0.05).

Based on scanning electron microscopy and RNA-seq data, 15 genes of interest including genes associated with extracellular matrix remodeling (TIMP3, MMP2, MMP3, and CTGF), energy metabolism (PPARγ and SLC2A1), ovarian steroidogenesis (StAR, CYP11A1 and LHCGR) and 6 top differently expressed genes (CEBPB, CXCL12, ANGPT2, IGFBP3, AREG and BBOX1) were selected to be validated by QRT-PCR. As shown in Figure 6, 6 genes (CEBPB, CXCL12, StAR, MMP2, CYP11A1, and LHCGR) were significantly up-regulated, and 9 genes (PPARγ, SLC2A1, TIMP3, MMP3, CTGF, ANGPT2, AREG, BBOX1, and IGFBP3) were significantly down-regulated in aging GCs, which were consistent with the RNA-seq data. These results indicated that RNA-seq generated a reliable dataset for the transcriptome analysis of aging GCs.

## DISCUSSION

GCs surrounding the oocyte are defined as the immediate ovarian microenvironment, thus, the processes of GCs required for supporting oocyte development may be the underlying targets for endocrine perturbations associated with aging [32]. Although several studies have established the relationship between poor oocyte quality and GC dysfunction in human and animal models [12, 33, 34], the effects of aging on physiological functions in GCs and their molecular mechanisms have not been widely examined. In this study, we simulated the aging process by extending the culture time, which has been suggested to closely mimic the process of aging [35, 36].

RNA-seq is a powerful tool for characterizing and quantifying the transcriptomes. In the present study, transcriptome analysis revealed that 3411 genes were differentially expressed; 2193 genes were up-regulated and 1218 genes were down-regulated during *in vitro* aging in GCs. KEGG pathway analysis revealed that 25 pathways including pathway in cancer (128 DEGs), PI3K-Akt signaling pathway (114 DEGs), focal adhesion (96 DEGs), proteoglycans in cancer (93 DEGs), and cAMP signaling pathway (71 DEGs) were significantly changed (*P* < 0.01). Based on these results, we believe that these differentially expressed pathways may be the key pathways that affect GC functions during aging.

Aging contributes to processes such as metabolic dysfunction, DNA damage, and reactive oxygen species (ROS) generation and can be considered as the progressive failure of balance in the body, resulting in impaired functions, health decline, and diseases [37-39]. As described above, aging can reduce lipid metabolism in GCs. Lipids are essential for physiological processes such as the maintenance of membrane integrity [37]. ROS can cause lipid peroxidation, which is closely associated with impaired mitochondrial function and changes in lipid metabolism. In this study, we analyzed the extracellular ultrastructure of the aging GCs by scanning electron microscopy. The results showed that membrane integrity was significantly impaired in aging GCs. The membranes of aging cells were rough and had many cavitations. In addition, the formation of filopodia was reduced (Figure 1). It is well-known that polyunsaturated phospholipids are the major components of the membrane, ROS-induced lipid peroxidation can damage these lipids and reduce membrane fluidity [40], which may explain the reduced formation of filopodia in this study. Membrane fluidity also modulates the functions of membrane proteins, such as ion channels and transporters, as well as various types of membrane receptors [41-46]. Even slightly changes in membrane fluidity may cause cellular dysfunction or induce pathological processes [47, 48]. Similarly, a loss of filopodia has been observed in ROS-overexpressing human breast cancer cells and lung cancer cells [49, 50], while the suppression of ROS was found to enhance the formation of filopodia [51]. Taken together, aging could up-regulate ROS, leading to decreased mitochondrial function, and disruption of membrane integrity and fluidity *via* lipid peroxidation, which may affect GC functions and result in low fertility in elderly women.

In addition to the cell membrane, the extracellular matrix (ECM) was also affected by aging. The ECM is a collection of molecules secreted by cells. Because of its diverse nature and composition, the ECM has many functions, such as providing support, segregating tissues from one another, and regulating intercellular communication. The ECM is vital for supporting ovarian follicle growth and maintaining its function, and it is involved in follicle development, cell migration, proliferation, and growth. In the present study, RNA-seq analyses showed that the expression levels of genes related to the ECM were significantly altered in aging GCs. For example, connective tissue growth factor (CTGF), also known as CCN2, is a cysteine-rich ECM protein that plays important roles in a variety of cellular functions, including cell proliferation, differentiation, apoptosis, migration adhesion, and ECM remodeling [52]. Indeed, CTGF ovarian conditional knockout mice were found to exhibit multiple reproductive defects and severe sub-fertility [53]. In our study, the decrease in CTGF was confirmed by QRT-PCR. Similarly, other studies have also reported the aging-related down-regulation of CTGF and organ dysfunctions [54].

ECM metabolism (including remodeling, homeostasis and turnover) is primarily regulated by matrix metalloproteinases (MMPs) and their endogenous inhibitors, tissue inhibitors of metalloproteinase (TIMPs) [55]. For the precise remodeling of the ECM, a delicate balance must exist between the activities of MMPs and TIMPs. Situations in which this control is lost may lead to pathological conditions [55]. Based on our results, TIMP3 expression was significantly attenuated in aging GCs. MMP2, MMP15, MMP23B and MMP17 were up-regulated, and MMP1, MMP3, and MMP16 were down-regulated. These results suggest that the balance of MMPs and TIMPs may be disrupted in aging GCs. However, thus far few studies have investigated the expression patterns and functions of MMPs and TIMP3 in aging GCs.

Disturbances in metabolism can also impair GC functions in particular, a failure in energy metabolism would be fatal. It is well-documented that glucose is a major source of metabolic energy for all mammalian cells [56] and is primarily transported across the plasma membrane through glucose transporters (GLUTs), also known as solute carrier family 2 (SLC2) [57, 58]. Among these transporters, GLUT1 (also known as SLC2A1) is considered as the predominant isoform [59]. The deregulated expression of GLUTs has been implicated in several pathological processes. For example, up-regulated GLUTs increase glucose uptake and accelerate tumor cell growth, resulting in poor survival [60-63]. GLUT1 overexpression is also closely related to glomerulosclerosis development [64] and results in the excess production of ECM proteins [65]. Accordingly, the inhibition of GLUT1 could significantly reduce cell viability and proliferation [66, 67], and contribute to an anticancer effects [68]. Interestingly, the knock down of GLUT1 expression can result in low CTGF expression [69], this finding is in agreement with our results. According to the RNA-seq and QRT-PCR results in this study, the expression of GLUT1 was significantly decreased in GCs during aging. These results suggest that aging-induced GLUT1 down-regulated and glucose uptake suppression are factors that could impair GC functions.

GLUT1 expression is regulated by transcriptional factors that bind to the promoter sequence. Various studies have demonstrated that peroxisome proliferator pctivated peceptor-γ (PPARγ) is a key regulator of GLUT1 expression [70-73]. The relationship between ROS and PPARγ has been determined previously; for example, when treated with organophosphate compounds, salmon liver cells showed decreased PPARγ expression and increased ROS production [74], which were restored to normal levels following antioxidant treatment [75]. Moreover, aging was found to suppress PPARγ in aged mouse model [76]. The Klotho beta (KLB) gene, originally identified as an aging-suppressor gene, could promote cell proliferation and differentiation; the overexpression of KLB has been reported to significantly enhance the expression of PPARγ [77]. In our study, both PPARγ and KLB were significantly decreased in aging GCs. Therefore, we believe that aging/ROS-KLB-PPARγ-GLUT1 signaling pathway dysfunction maybe involved in aging GCs.

As previously reported, a characteristic of aging GCs is premature luteinization [78, 79]. CCAAT/enhancer binding proteins beta (CEBPB) is an essential regulator of luteinization [80]. CEBPB belongs to the CEBP basic-leucine zipper transcription factor family. It has been reported to increase following ovulations and hCG stimulation in porcine GCs [81, 82]. CEBPB has also been shown to participate in steroidogenic acute regulatory protein (StAR) regulations by interacting with an E-box in the stimulation of PKC/forskolin or gonadotropin/cAMP activation in GCs and luteal cells [83]. Furthermore, CEBPB plays an important role in cAMP-induced CYP11A1 and LHCGR expression [80, 84]. Based on our data, CEBPB was markedly increased in GCs during aging, accordingly, LHCGRR, StAR and CYP11A1 were increased. These results convert with that in elderly women and mares [85, 86]. These results suggested that CEBPB may play important roles in GC aging; however, further studies are required to confirm its function.

Insulin-like growth factor binding protein 3 (IGFPB3) has dual functions in regulating IGF activity; the soluble form of IGFBP3 inhibits the activity of IGF1 by suppressing receptor binding, whereas surface-associated IGFBP3 enhances the growth-promoting effects of IGF1 [87]. Aging has been reported is a factor that decreases IGFBP3 expressions in human columns cells [88], and low IGFBP3 levels always result in the arrest of oocyte or embryo development [89]. In addition, angiopoietin 2 (ANGPT2) is an angiogenic factor that plays an important role in the modulation of angiogenesis and vasculogenesis; thus, it is a vital factor in corpus luteum development. ANGPT2 expression has been reported to be increased by PKC and PKA activators in GCs [90] and in the early luteal phase [91]. Moreover, ANGPT2 expression was found to be increased by hCG in leydig cells [92], and decreased in aging endothelial cells [93]. In the present study, IGFBP3 and ANGPT2 were the two most down-regulated genes in aging GCs and were confirmed by QRT-PCR. These results revealed the involvement of both the IGFBP3 and ANGPT2 in aging in GCs *via* the suppression of oocyte development and angiogenesis.

In conclusion, we used RNA-seq to examine the gene expression profiles of aging GCs *in vitro*. Several DEGs were detected and the possible pathways were predicted by KEGG analyses. These results provide a foundation for further studies on aging. However, the precise functions of these pathways remain unknown; thus further studies are required to confirm our results.

## MATERIALS AND METHODS

### Granulosa cells isolation, cultivation and treatment

Ovaries of prepubertal gilts aged 170-180 days were obtained from a local slaughterhouse and transported to the laboratory in a vacuum thermos flask in sterile physiological saline at 37 °C within 2 h of isolation. After ovaries were thoroughly washed with sterile physiological saline at 37 °C, follicular fluid and GCs were aspirated from follicles with 6-8 mm in diameter that contained clear follicle fluid using 10 mL syringe with 20-gauge needle. The cells were then transferred to a 15 mL centrifuge tube, and 1 mL of 0.25% trypsin was added to digest cell lumps. Following incubation at 37 °C for 3-5 min to disperse clumps of cells, 1 mL of 10% fetal bovine serum (FBS)-supplemented Dulbecco’s modified Eagle’s medium/Ham’s F-12 nutrient mixture (DMEM/F12, without phenol red) was added to the tube to terminate trypsin digestion. The cells were then centrifuged at 800×g for 15 min to be precipitated and then washed twice with phosphate-buffered saline (PBS). Cell density was adjusted to 5 × 10^6^ cells in a 6 cm dish, in 2 mL of DMEM/F12 containing 10% FBS and 1 ng/mL porcine pituitary FSH (F2293, Sigma), with the supplementation of FSH, it can stimulates GCs growth and prevented from luteinization *in vitro*, and also inhibits the apoptosis [94]. The cells were incubated under a humidified atmosphere containing 5% CO_2_ at 37 °C for 24 h, and then washed with PBS to remove the unattached cells.

After an initial 24 h culture as described above, the cells were divided into two groups (control group and *in vitro* aging group) randomly. Then, the culture media were replaced with fresh DMEM/F12 containing 10% FBS, 0.1 μM 19-hydroxyandrostenedione (Sigma) and 1 ng/mL porcine pituitary FSH for further 3 h (control group) and 48 h (*in vitro* aging group) culture, respectively. Culture medium in aging group was changed at 24 h to diminish the negative effect of metabolic byproducts [95].

### Ultrastructure observation by scanning electron microscope

GCs were cultured on coverslips and treated as described before. Then, the cultured GCs were fixed according to scanning electron microscopy (SEM) methods. Briefly, samples were fixed for 1 day in 2.5 % glutaraldehyde in 0.1 M phosphate buffer and rinsed briefly in distilled water at room temperature. Then the specimens were dehydrated in a series of graded concentrations of ethanol (50 %, 70 %, 80 %, and 90 % for 15 min each, finally at 100 % ethanol for 30 min, 3 times) and critical point dried in a K850 dryer (Quorum, UK). The dried specimens were mounted on metal stubs, coated with gold film (10 nm) using 108Auto Sputter Coater (Cressington, UK) and observed using a scanning electron microscope (Carl Zeiss, EVO LS10, DE) with an accelerating voltage of 10 kV.

### RNA quantification, library construction and sequencing analysis

After treatment, cells were harvested using TRIzol reagent (Invitrogen, USA). Cell samples were collected from 3 separated experiments. After the total RNA were obtained, the degradation, DNA contamination and purity were monitored by 1.5% agarose gel and Nanodrop 2000 spectrophotometer (Thermo Fisher, Wilmington, DE), respectively. RNA integrity was assessed using RNA Nano 6000 Assay Kit and Agilent Bioanalyzer 2100 system (Agilent Technologies, CA, USA).

The 6 RNA-seq libraries (3 RNA-seq libraries for Control and 3 RNA-seq libraries for aging GCs) were generated using an Illumina TruSeqTMRNA Sample Preparation Kit (Illumina, San Diego, USA) as the manufacturer’s instructions. The libraries were sequenced by pair-end sequencing on an Illumina HiSeq 2000 platform and 100 bp paired end reads were generated. The base quality distribution of different RNA-seq library was summarized. Raw data (raw reads) of fastq format were firstly processed through in-house perl scripts. Clean data (clean reads) were obtained by removing reads containing adapter, reads containing ploy-N and low quality reads from raw data. At the same time, Q30, GC-content and sequence duplication levels of the clean data were calculated.

### Identification of differentially expressed genes

Based on RNA sequencing, gene expression levels were measured as the number of reads per kb of exon regions per million mapped reads (RPKM). DEGs were detected with the R package DEGseq software. A corrected *P*-value of 0.05 and a |log2 (fold change)| of 1 were set as the threshold for statistically significant differential expression. For the genes that were differentially expressed in aging GCs, the unigene numbers were converted to Entrez Gene ID numbers using the online g: profile conversion tool (http://biit.cs.ut.ee/gprofiler/).

### GO enrichment analysis and KEGG pathway enrichment analysis

To facilitate elucidating the biological functions of the differentially expressed genes (DEGs), Gene Ontology (GO) analysis was usually performed. The enrichment analysis of the DEGs was implemented using the topGO-R software packages. GO annotations were according to NCBI (http://www.ncbi.nlm.nih.gov), UniProt (http://www.uniprot.org/) and the Gene Ontology (http://www.geneontology.org/). GO terms with corrected *P* values less than 0.05 were considered significantly enriched [96]. Pathway analysis software KOBAS was used to test the statistical enrichment of differentially expressed genes according to the Kyoto Encyclopedia of Genes and Genomes (KEGG) database (http://www.genome.jp/kegg). A corrected *P*-value of 0.05 was set as the threshold to identify significantly different pathways [96] and *P-*values were adjusted by Benjamini-Hochberg method [97].

### Validation of RNA-Seq data by quantitative real time RT-PCR assays

Real-time quantitative polymerase chain reaction was performed to quantify the mRNA expression levels of GAPDH, PCRs were carried out in a 20 μL reaction volume containing SYBR Green I Master Mix (TaKaRa, China) and 100ng cDNA template. An ABI 7500 system (Applied Biosystems; Foster City, CA, USA) was used to detect the amplification products. Upon completion of the real-time qPCR, threshold cycle (Ct) values were calculated by ABI 7500 software V.2.0.6 (Applied Biosystems; Foster City, CA, USA). The levels of gene expression were expressed in the comparative ΔCt method using the formula of 2^-ΔΔCt^ and normalized to the expression levels of the GAPDH internal housekeeping gene. All the primers used in this study as shown in Supplementary Table 1. Three separate experiments were performed on different cultures, and each sample was assayed in triplicate.

### Statistical analysis

Differences gene expressions in GCs between treated and control groups, were analyzed by one-way analysis of variance (ANOVA). Results are presented as the mean ± SEM of at least three separate experiments performed on different cultures. All statistical analyses were performed with SAS software Version 8.01 (SAS Institute Inc.; Cary, NC, USA). *P* < 0.05 was considered significantly different.

## SUPPLEMENTARY MATERIALS AND TABLE












