# Supplementary material for: Systemic analysis of gene expression profiles in porcine granulosa cells during aging
Source: Oncotarget. 2017 Oct 10;8(57):96588–603. doi: 10.18632/oncotarget.21731 (PMC5722506; doi:10.18632/oncotarget.21731)
Supplement: Supplementary file 1 [file oncotarget-08-96588-s001.pdf]

Systemic analysis of gene expression profiles in porcine granulosa cells during aging

SUPPLEMENTARY MATERIALS

Supplementary Table 1: Primers used in this study

Gene	Primer sequences (5′-3′)	Length (bp)
GAPDH	F: GTCGGTTGTGGATCTGACCT<br>R: TTGACGAAGTGGTCGTTGAA	207
PPARγ	F: GCTGACCAAAGCAAAGGC<br>R: ACGGAGCGAAACTGACACC	189
SLC2A1	F: GCTTCCAGTATGTGGAGCAA<br>R: AAGCAATCTCATCGAAGGTC	132
MMP2	F: TACACCTATACCAAGAACTTCCG<br>R: TGTCCGCCAGATGAACCG	216
MMP3	F: GACGGGGAAGCTGGATTCTA<br>R: CCAGGTGCATAGGCATGAG	339
CTGF	F: GGAAATGCTGCGAGGAGTGG<br>R: CGTGTCTTCCAGTCGGTAAGC	89
TIMP3	F: CTCTGCAACTCCGACATCGTGAT<br>R:CAGCAGGTACTGGTACTTGTTGAC	210
CEBPB	F: GTGGCGCCGGCAAAACTT<br>R: GAGGGGGCAGGAGGAGAGGCAGAG	203
IGFBP3	F: AAATGGAGGACACGCTGAAC<br>R: TACTTATCCACGCACCAGCA	157
CXCL12	F: GCCAGAGCCAACATCAAGCA<br>R: AAACATCCCGCCGTCCTCA	250
AREG	F: CCGTGGTGCTGTCACTCTTGATC<br>R: CGAGGACATCTCACTTCTGGAGGT	149
ANGPT2	F: AAAGTTGCTGCAGGGAAAGA<br>R: TCACAGCTCAGAGCGAAGAA	191
BBOX1	F: GTGCCGAAAGCTCAAGGAAAAA<br>R: CTCTGCCGGCCGTGAAGTAAC	342
CYP11A1	F: TCCCATTTACAGGGAGAAGCTCG<br>R: GGCTCCTGACTTCTTCAGCAGG	182
STAR	F: CATTACCATCTACTCCCAGC<br>R: AACCCGTATCTTTCTTGTCAG	109
LHCGR	F: CATGGCACCGATCTCTTTCT<br>R: CGGAATGCCTTTGTGAAAAT	150

For Supplementary Files see in Supplementary Files.
